# Corrosive injury of the trachea in children

**DOI:** 10.1002/ccr3.2395

**Published:** 2019-09-11

**Authors:** Pierre Goussard, Lunga Mfingwana, Julie Morrison, Zane Ismail, Riegart Wagenaar, Jacques Janson

**Affiliations:** ^1^ Department of Paediatrics and Child Health Faculty of Medicine and Health Sciences Stellenbosch University and Tygerberg Hospital Cape Town South Africa; ^2^ Department of Cardiothoracic Surgery Faculty of Medicine and Health Sciences Stellenbosch University and Tygerberg Hospital Cape Town South Africa

**Keywords:** balloon dilatation, corrosive injury, tracheal injury, tracheal resection, tracheal stenosis

## Abstract

The secondary injury may present weeks to months after the initial insult and repeat bronchoscopy, and long‐term follow‐up is required for the respiratory complications of CSI.Ingestion of caustic fluid may cause severe tracheal stenosis. Repeated airway dilatation may be a lifesaving intervention until such point that surgery can be performed.

## 1 INTRODUCTION

It is fairly uncommon for children to present to the medical emergency room with a history of ingestion of a caustic substance, yet this phenomenon has serious medical implications for the patient that the managing pediatrician/surgeon should be alerted to. Caustic substance ingestion (CSI) accounts for 5‐518/100 000 cases per year in children.^1^ CSI remains a public health issue in low‐ and middle‐income countries.^2^

Most CSI cases are accidental in children. Depending on the type of ingested substance, the amount ingested and the time of tissue exposure, patients experience different types of injuries of varying severity. Injury caused by alkali‐based substances causes a different injury pattern to or acid‐based substances.

There has been a considerable amount of literature published on CSI resulting in injury to the gastrointestinal tract and the upper airways in children, however, limited information is available on tracheal injuries suffered after CSI in children.^3^, ^4^, ^5^ The epiglottis and arytenoids are affected by the caustic bolus passing through the hypopharynx. The patients may need intubation and ventilation due to the upper airway injury and swelling.

We report two cases of caustic ingestion who presented with respiratory symptoms, requiring intubation, ventilation, and multiple subsequent endoscopic procedures to relieve their symptoms. Consent was given by both children's parents for publication of the cases.

## 2 CASE 1

A 10‐year‐old male, who was previously well, was found hypothermic, bradycardiac, with an Glasgow coma scale of nine, with difficulty breathing at home after intentionally ingesting an unknown amount of undiluted glyphosate‐based herbicide solution.

He was intubated with an uncuffed size six endotrachael (ET) tube (patient *weight *40 kg) and ventilated. No gastric lavage was done and no gastroscopy or bronchoscopy was performed. He was ventilated for 2 days and discharged from hospital after another 3 days.

Two weeks after discharge he presented to hospital with noisy breathing and hemoptysis. He was admitted with upper airway obstruction and monophonic wheezing. The patient was started on adrenaline nebulization, oral prednisone, and Amoxicillin/clavulanic acid treatment.

**Figure 1:**
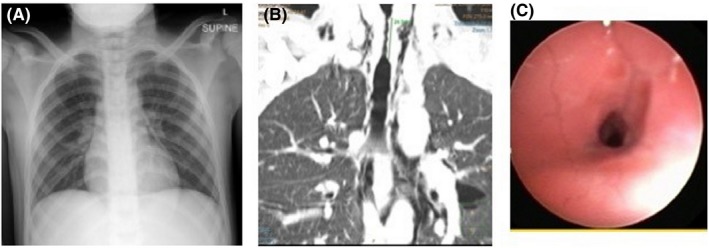
A, Chest X‐ray demonstrating narrowing of the proximal trachea and pneumomediastinum. B, Coronal chest CT‐scan reconstruction: Extensive narrowing of the proximal trachea, starting just below the subglottic area. Air is visible in the mediastinum. C, bronchoscopy picture: severe narrowing of the trachea just below the subglottic area

A chest X‐ray (Figure 1A) showed long segment narrowing of the proximal trachea, with a pneumomediastinum. The lung function test showed a fixed obstructive pattern. A barium swallow examination showed no abnormalities.

Due to worsening airway obstruction symptoms, a bronchoscopy was performed. Severe stenosis of more than 75% of the proximal trachea was found (Figure 1C). The stenosis was located in the subglottic region, 2 cm below the glottis, and extended over a distance of 2 cm. This stenosis was dilated with rigid dilators to a size of 32 F.

A thoracic CT scan was subsequently preformed, in order to demonstrate the extent and severity of the stenosis to aid in planning for surgical dissection. The area of stenosis was identified as being 2.4 cm long, with an irregular appearance (Figure 1B).

The patient required a number of further dilatations of the trachea, until the active inflammation had resolved and was amenable to surgical intervention. Short segment tracheoplasty was done, with a good final outcome. The patient remained asymptomatic after surgery.

## 3 CASE 2

A 4 ½‐year‐old girl presented to a local clinic with a history of accidental ingestion of approximately ~15 mL of hydrogen peroxide/peracetic acid. The patient was found by her mother, vomiting profusely a very offensive liquid. On arrival, the patient was noted to be in severe respiratory distress, with overwhelming oral secretions, a biphasic stridor and respiratory failure.

The patient was subsequently intubated with an uncuffed size four ET tube (patient weight 17 kg) by the resident anesthetist, who reported the true vocal cords to be swollen. Dexamethasone and pantoprazole were added.

Bronchoscopy and gastroscopy were preformed on day 5 of ventilation, with the intention to extubate her in theater. The initial bronchoscopy showed inflammation of the supraglottic structures, with severe edema around the ET tube and grade II circumferential burns in the esophagus. The patient remained intubated and on oral steroids and was extubated 7 days after the initial bronchoscopy.

On extubation in bronchoscopy theater, there was still inflammation of the supraglottic structures, with fibrous granulation. Normal vocal cord movement was observed, and the trachea appeared normal. The patient was kept on steroids, nasogastric feeds, and anti‐reflux medication for one further week, with marked improvement of her clinical symptoms.

**Figure 2:**
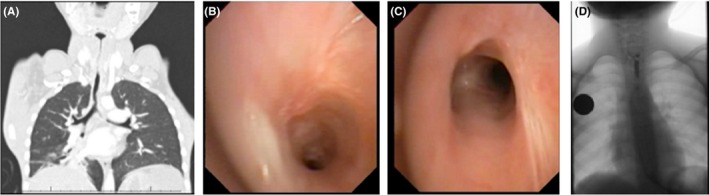
A, Coronal chest CT‐scan reconstruction: Demonstrating two levels of narrowing of the trachea, with a very narrow proximal part and irregular shape of most of the trachea. B and C, Bronchoscopy images of the proximal and distal areas of narrowing, also demonstrating severe mucosal injury. D, Contrast swallow study: Severe short segment narrowing of the esophagus

Three weeks postdischarge, the patient was readmitted to hospital with worsening monophonic wheezing. Repeated bronchoscopy now showed a funnel‐shaped multi‐level tracheal obstruction. There were two distinctive areas of tracheal stenosis, one in the proximal trachea and the second one just superior to the carina (Figure 2B and C). The proximal stenosis occluded appromiately 75% of the lumen, and the distal stenosis resulted in approximately 50% luminal occlusion. There was mucosal injury involving the entire length of the trachea with fibrotic scarring developing. A CT scan of the chest demonstrated multiple levels of tracheal stenosis, with a very abnormal tracheal caliber. Both the proximal and the distal stenoses were identified on imaging (Figure 2A) The child received multiple airway dilatations with both balloon and rigid dilators. The distal area of stenosis responded well to balloon dilatation. The proximal area of stenosis was significantly more difficult to dilate. After 20 dilatations, the proximal stenosis had stabilized to 4.5 mm, and the distal tracheal stenosis has significantly improved to 5 mm. The patient also has significant esophagael stenosis that requires repeated dilatation (Figure 2D).

## 4 DISCUSSION

Caustic ingestion in children presenting with both upper and lower respiratory symptoms may requiring intubation and multiple subsequent endoscopic procedures to relieve their symptoms.

The mortality of corrosive injury is lower in children than adults, owing to the fact that children are more likely to ingest harmful substances accidentally, as opposed to attempting suicide.^3^, ^6^, ^7^^.^ There is a bimodal fashion of presentation, with the first peak in the 1‐ to 5‐year‐old age group, as young children are more likely to ingest caustic substances, either accidentally or out of curiosity. The second peak is found in adolescents, with many of these ascribed to intentional suicide attempts.

Corrosive injury to the upper airway and the esophagus is well described. Corrosive injury to the trachea has been described in adults, but limited pediatric data is available. 8 Most acquired airway injuries in children are related to intubation. 9 ^.^ Approximately 1%‐2% of CSI cases in all age groups result in tracheal stricture formation.^10^

The identified risk factors for CSI are environmental factors and include low levels of parental education, overcrowding, lack of supervision, low‐socioeconomic status, and neurodevelopmental and behavioral disorders.^2^, ^11^

The pathophysiology of CSI injury varies depending on whether the ingested substance is acidic or alkaline.

Ingestion of an alkali‐based substance is associated with minimal irritation to the oral cavity, thus large volumes are ingested. Naturally, alkali substances are thicker, and this leads to longer exposure time after ingestion, causing progressive injury with increased depth of injury and stricture formation.^12^ Alkaline substances cause tissue injury by liquefactive necrosis, involving solubilization of proteins, saponification of fats and eventually resulting in cell death.^10^, ^13^

Significant airway injury can appear in a very short time, due to the substantial risk of soft tissue injury which occurs within minutes after ingestion, resulting in mucosal contact with the alkaline substance. Tissue swelling may persist for up to 48 hours, after which necrotic tissue is replaced by granulation tissue. 14

The longer the contact time with the alkaline substance, the higher the risk for increased depth of injury and stricture formation.^14^

Acidic substances induce a burning sensation, with subsequent pain immediately after contact with the oral mucosa, and accordingly, the volumes ingested traditionally tend to be small. Acid causes coagulation necrosis, which results in denaturation of the superficial protein layer and eschar formation. This may protect against further damage, but the eroded mucosa is replaced by granulation tissue as it matures, which may cause luminal obstruction and has the potential risk of perforation and hemorrhage.^10^, ^13^

Severe systemic symptoms and complications have been reported following caustic ingestion, and these include hemolysis, disseminated intravascular coagulation, renal failure, liver failure, perforated viscus, peritonitis, mediastinitis, and death.^15^

Corrosive injury can progress weeks to months after the original insult.^3^, ^16^ In both cases presented, there was progression of airway injury days to weeks after the initial CSI. In the second case the trachea had a normal appreance during the original bronchoscopy, macroscopically, and the CSI subsequently progressed to tracheal stenosis. In this case, the contrast study did show gastro‐esophageal reflux, which might have contributed to the late onset of tracheal injury.

Management of CSI cases is complicated. Surgery cannot be done early during the course of the postinjury period, because of the risk of ongoing fibrosis. The airway needs to be dilated until the process of healing and fibrosis has settled. Endoscopic laryngotracheal dilation can be done both with rigid dilators and with inflatable balloons. Hautefort et al concluded that tracheal balloon dilation has become the first therapeutic option in acquired subglottic stenosis (SGS) of all grades, stenosis secondary to prior tracheal reconstructive surgery and in selected cases of congenital SGS, reducing the need for an open approach in 70%‐80% of cases.^15^

Patient management might be complicated by the presence of esophageal strictures, as was the case in the second case described. Dilatation of both the airway and esophagus is not without risk, and the reported complications include iatrogenic perforation, pneumothorax, pneumomediatinum, and swelling of the airways.^17^ The narrower the stenosis, the higher the risk of complications during these interventions.

In case one end‐to‐end tracheoplasty was done after 6 weeks, due to the short nature of the stenosis. In case two, the whole length of the trachea was involved with two areas of significant stenosis. The distal stenosis responded well to balloon dilatation, which had to be repeated three times. The proximal stenosis needed rigid dilatation, which needed to be repeated every 2‐3 weeks. The airway in this case stabilized after 20 dilatations, however, the patient still required frequent endoscopy investigation for monitoring of the disease process.

Prevention is still the mainstay of managing CSI. Improper storage of caustic materials continues to be a major risk for caustic ingestion. Reducing the occurrence of CSI is solely embedded in improved parent and community education.

We would advocate both early upper airway and lower airway endoscopy, as it is important to determine if injury has occurred, the extent of the injury and to guide extubation. The endotracheal tube needs to be removed to evaluate the trachea for injury. Follow‐up bronchoscopy has to be performed 1‐2 weeks after CSI to monitor disease progression and enable early dilatation of the trachea. Subsequently regular clinical follow‐up is indicated, with repeat bronchoscopy when indicated for the first 6 months after the insult. Corticosteroid therapy is prescribed in children with airway symptoms; however, most studies indicate that corticosteroids are not beneficial for the prevention of stricture formation.^16^, ^18^, ^19^, ^20^

## 5 CONCLUSION

The ingestion of caustic substances still poses a serious risk to children. The gastrointestinal injuries associated with CSI have been well studied, with early and long‐term complications well described, however, secondary respiratory injury has been under‐reported in the literature, and the entity is often overlooked. The injury may progress weeks to months after ingestion and repeat bronchoscopy is indicated. Ingestion of caustic fluid may cause severe tracheal injury in children. Repeated airway dilatation may be a lifesaving measure until such time that surgery can be performed.

## CONFLICT OF INTEREST

None declared.

## AUTHOR CONTRIBUTIONS

Pierre Goussard: Responsible for clinical management of the patients and lead clinician. Main author of the paper. Lunga Mfingwana and Julie Morrison: Responsible for clinical management of the patients and collecting the information. Zane Ismail, Riegart Wagenaar, and Jacques Janson: responsible for the surgical management of the patients.
